# Quality of life and symptom intensity over time in people with cancer receiving palliative care: Results from the international European Palliative Care Cancer Symptom study

**DOI:** 10.1371/journal.pone.0222988

**Published:** 2019-10-09

**Authors:** Mariëtte N. Verkissen, Marianne J. Hjermstad, Simon Van Belle, Stein Kaasa, Luc Deliens, Koen Pardon

**Affiliations:** 1 End-of-Life Care Research Group, Department of Family Medicine and Chronic Care, Vrije Universiteit Brussel (VUB) & Ghent University, Brussels, Belgium; 2 European Palliative Care Research Centre (PRC), Department of Oncology, Oslo University Hospital, and Institute of Clinical Medicine, University of Oslo, Oslo, Norway; 3 Department of Medical Oncology, Ghent University Hospital, Ghent, Belgium; 4 European Palliative Care Research Centre (PRC), Department of Cancer Research and Molecular Medicine, Faculty of Medicine, Norwegian University of Science and Technology (NTNU), Trondheim, Norway; 5 Cancer Clinic, St. Olavs Hospital, Trondheim University Hospital, Trondheim, Norway; 6 Department of Public Health and Primary Care, Ghent University, Ghent, Belgium; Iranian Institute for Health Sciences Research, ISLAMIC REPUBLIC OF IRAN

## Abstract

**Background:**

People with advanced cancer experience multiple symptoms during their illness trajectory, which can fluctuate in intensity.

**Aim:**

To describe the course of self-reported quality of life, emotional functioning, physical functioning and symptom intensity over time in cancer patients receiving palliative care.

**Design:**

Longitudinal study with monthly assessments, using the EORTC QLQ-C15-PAL. Data were analysed (1) prospectively, from baseline to ≥8-month follow-up; and (2) retrospectively, by taking death as index date and comparing results from three cross-sectional subsamples at different stages of illness (time to death ≥6, 5–3 and 2–0 months). Linear mixed models were calculated.

**Setting/participants:**

A total of 1739 patients (mean age 66, 50% male) from 30 palliative care centers in 12 countries were included.

**Results:**

In prospective analyses, quality of life, functioning and symptoms–except nausea/vomiting–remained generally stable over time. In retrospective analyses, patients 2–0 months before death reported significantly lower quality of life and physical functioning scores than those 5–3 months before death, who in turn scored lower than those ≥6 months before death, suggesting progressive decline. Emotional functioning remained initially unchanged, but decreased in the last months. Pain, fatigue and appetite loss showed a stable increase in intensity towards death. Dyspnea, insomnia and constipation increased from 5–3 to 2–0 months before death. Nausea/vomiting only increased when comparing those ≥6 months before death with those 2–0 months before death.

**Conclusion:**

While the prospective approach showed predominantly stable patterns for quality of life, functioning and symptom severity throughout study duration, retrospective analyses indicated that deterioration was already apparent before the terminal phase and accelerated close to death. Our findings support the importance of early symptom identification and treatment in this population, and highlight the need for further studies to explore what characterizes those with either lower or higher symptom burden at different time points towards death.

## Introduction

Due to aging and more effective treatments, more people are living longer after being diagnosed with cancer, even those with progressive, incurable cancer. This may have detrimental effects on physical and emotional health [1,2]. Symptoms such as anxiety, depressed mood, pain, fatigue, dyspnea and appetite loss [3] can significantly impact quality of life and patients’ ability to carry out daily activities [4–6]. Therefore, high-quality palliative cancer care requires optimal symptom management across the disease trajectory and towards the end of life, when patients with advanced cancer may experience worsening symptom burden and evident functional decline [7–9].

Understanding how quality of life (QoL), emotional functioning (EF), physical functioning (PF) and symptoms progress over time is important, since it can help healthcare professionals working in palliative care achieve the best possible outcomes for patients at any point along the course of the advanced illness. However, although previous studies have described QoL, functioning and symptom burden in cancer patients in a palliative care setting, these studies were mostly cross-sectional or, if prospective, limited in follow-up. Describing changes in these variables over time requires the use of a design involving repeated registrations over an extended period of time.

In the present study, we first looked prospectively at a large international sample of people with cancer enrolled in palliative care, to address the following research questions: (1) how do QoL, EF and PF evolve over time, and (2) how does the intensity of cancer-related symptoms evolve over time? Secondly, we addressed these research questions retrospectively; therefore, we focused on those participants who had passed away during the study period, and examined whether QoL, functioning and symptoms differed significantly between three cross-sectional subsamples of deceased patients based on time to death (≥6, 5–3, and 2–0 months).

## Methods

### Study design and setting

We used data from the multi-center longitudinal European Palliative Care Cancer Symptom (EPCCS) study, which was conducted in 30 palliative care centers in 12 countries (10 European countries, Australia, and Canada). The study ran from April 2011 through October 2013. The participating centers were 24 hospital departments, four hospices, one nursing home, and one palliative home care service. Details of the study and participating centers can be found elsewhere [10].

### Study population

The study aimed to include a large number of palliative care cancer patients from different sites, with mixed cancer diagnoses and at various stages of their disease. Inclusion criteria were: advanced, incurable cancer confirmed through radiological, histological, cytological, or operative evidence; age ≥18 years; enrolled in a palliative care program; written informed consent; and eligible for at least one follow-up assessment after inclusion. Exclusion criteria were: being treated with curative intent; inability to comply with study procedures due to psychiatric disorders; severe cognitive impairment or language problems; imminent death; or inability to come for follow-up due to medical, social, or geographical reasons.

### Measurements

The data used in this paper were collected using a case report form on medical data completed by healthcare providers (HCP-CRF) and by participants’ self-report on health and symptoms (patient-CRF). Both CRFs were completed monthly (3–5 weeks) for a minimum of three months, or until death or study withdrawal.

The HCP-CRF consisted of a brief set of medical and treatment-related variables, e.g. primary cancer diagnosis, comorbidities, anti-cancer treatment, and medication. A retrospective recording of date of death was performed in each study center in February 2014, approximately six months after the last study inclusion.

The patient-CRF consisted of key socio-demographic characteristics, e.g. age, gender, marital status, living situation and education (collected at baseline), and questions on quality of life (QoL) and symptom intensity. The variables of interest in our analysis were overall QoL, emotional functioning (EF), physical functioning (PF) and cancer-related symptoms as assessed by the palliative care version of the European Organization for Research and Treatment of Cancer Quality of Life Questionnaire (EORTC QLQ-C15-PAL) [11,12]. The QLQ-C15-PAL consists of one item referring to overall QoL; an EF scale (four items: two extra items on depression from the full EORTC QLQ-C30 were added to the original two items); a PF scale (three items); a pain scale (two items); a fatigue scale (two items); and five single items (nausea/vomiting, dyspnea, insomnia, appetite loss, constipation). Patients responded to a four-point Likert scale from 1 (not at all) to 4 (very much), except for the item on overall QoL, which was rated on a seven-point numerical scale from 1 (very poor) to 7 (excellent).

Scores and scale scores were calculated following the EORTC QLQ-C30 Scoring Manual [13] and its addendum [14]. After standardization by linear transformation, scores range from 0–100. Higher scores on overall QoL and the functioning scales represent higher QoL and higher levels of functioning. Higher symptom scores indicate more severe symptoms.

### Data analysis

Statistical analyses were performed using IBM SPSS Statistics version 23.0. We handled missing data in the outcome variables according to the procedure outlined in the EORTC Scoring Manual [13]. If at least half of the items within a scale were completed, missing values were replaced with the average of the items that were present for the corresponding scale. If less than half of the items within a scale were answered, the scale score was defined as missing.

Linear mixed model (multilevel) analyses were conducted with repeated measures on patients nested in hospitals, and hospitals in countries. QoL, functioning and symptoms were the outcomes. Since assessments were completed at various (non-equidistant) time points, a time variable was included in each model (in months since baseline or months prior to death, depending on the analysis). Akaike’s Information Criterion (AIC) [15] was used to select the most appropriate covariance structures to fit the data. All models included a random intercept and a random slope for time at the three levels.

For the prospective analysis, time points of patient assessments were rounded to the nearest whole month since baseline. Estimated mean scores for QoL, EF, PF and symptoms with 95% confidence intervals were calculated for each month. Because of small numbers of observations, months 8–14 were combined (≥8). Linear mixed models as described above were used to test whether mean scores for the different time points differed significantly from the baseline value. Bonferroni-Holm adjustment was applied to correct for the problem of multiple comparisons. Mann-Kendal tests were used to detect consistently increasing or decreasing (monotonic) trends.

For the retrospective analysis, we used death as index date. Time points of patient assessments were rounded to the nearest whole month prior to death. Estimated mean scores and 95% confidence intervals were calculated for three cross-sectional subsamples which were constructed based on time to death, namely ≥6 (group 1), 5–3 (group 2), and 0–2 months (group 3). Statistical differences in mean scores were evaluated between the three groups.

All analyses were two-tailed and *p*-values smaller than 0.05 were considered statistically significant. In those cases where Bonferroni-Holm adjusted *p*-values were calculated (prospective analysis), the experiment-wise error rate (EER) was set at 5%.

### Ethical considerations

The study was performed according to the Declaration of Helsinki and was registered in the ClinicalTrial.gov database (no. NCT01362816). Ethical approval was obtained at each site and all participants gave written informed consent prior to study start. The ethics committees/institutional review boards of following centers gave ethical approval for the study: Southern Adelaide Palliative Services, Adelaide, South Australia (Australia); Ghent University Hospital, Ghent (Belgium); Comprehensive Cancer Centre, Vratsa (Bulgaria); Cross Cancer Institute, Northern Alberta (Canada); The Edmonton Zone Palliative Care Program, Alberta (Canada); Rigshospitalet, Copenhagen (Denmark); Bispebjerg Hospital, Copenhagen (Denmark); Cancer Prevention Center (CPC), Tbilisi (Georgia); Fondazione IRCCS Istituto Nazionale dei Tumori, Milan (Italy); Hospital of Piacenza, Piacenza (Italy); Hospice Villa Speranza, Rome (Italy); Istituti Clinici di Perfezionamento Hospital, Milan (Italy); U.O. Complessa di Cure Palliative e Terapia del Dolore, Istituti Clinici di Perfezionamento Hospital, Milan (Italy); University of L’Aquila, L’Aquila (Italy); Arcispedale Santa Maria Nuova, Reggio Emilia (Italy); St. Olavs University Hospital, Trondheim (Norway); Oslo University Hospital, Oslo (Norway); Haraldsplass Deaconess Hospital, Bergen (Norway); Øya Community Hospital, Trondheim (Norway); Instituto Português de Oncologia Francisco Gentil, Lisbon (Portugal); Hospital Universitário Arnau de Vilanova, Lleida (Spain); Clínica Universidad de Navarra, Pamplona (Spain); Hospital Centro de Cuidados Laguna, Madrid (Spain); Institut Catala D’Oncologia, Barcelona (Spain); Cantonal Hospital, St. Gallen (Switzerland); Kantonsspital Graubünden, Chur (Switzerland); St Gemma’s Hospice, Leeds (United Kingdom); West Lothian Community Specialist Palliative Care Team, Edinburgh (United Kingdom); Nottingham University Hospitals NHS Trust, Nottingham (United Kingdom); Marie Curie Cancer Care Hospice, Glasgow (United Kingdom).

## Results

### Characteristics of the study population

The baseline sample consisted of 1739 people from 12 different countries. At study entry participants had a mean age of 65.9 years (SD = 12.4), and there was an even gender distribution (*Table 1*). The predominant diagnoses were cancer of the digestive (30.4%) and respiratory organs (19.8%) and breast cancer (16.5%). At inclusion, 41.4% were receiving chemotherapy, while 40.6% were not receiving any treatment. Most people were included at hospital palliative care units (46%) and general oncology departments (34.5%).

**Table 1:** Patient characteristics.

**Socio-demographic characteristics at baseline (*n* patients = 1739)**		Missing, *n* (%)
**Age, mean ± SD**	65.9 ± 12.4	1 (0.1)
**Gender, *n* (%)**		3 (0.2)
Male	865 (49.8)	
Female	871 (50.2)	
**Country, *n* (%)**		0 (0.0)
Australia, 1 site (AU)	35 (2.0)	
Belgium, 1 site (WE)	101 (5.8)	
Bulgaria, 1 site (EE)	31 (1.8)	
Canada, 2 sites (AM)	94 (5.4)	
Denmark, 2 sites (NE)	104 (6.0)	
Georgia, 1 site (EE)	19 (1.1)	
Italy, 7 sites (SE)	605 (34.8)	
Norway, 4 sites (NE)	249 (14.3)	
Portugal, 1 site (SE)	62 (3.6)	
Spain, 4 sites (SE)	233 (13.4)	
Switzerland, 2 sites (CE)	72 (4.1)	
United Kingdom, 4 sites (WE)	134 (7.7)	
**Clinical characteristics at baseline (*n* patients = 1739)**		Missing, *n* (%)
**Primary cancer diagnosis, *n* (%)**		0 (0.0)
Digestive organs	528 (30.4)	
Respiratory organs	345 (19.8)	
Breast	287 (16.5)	
Male genital organs	129 (7.4)	
Gynaecological	103 (5.9)	
Urinary	79 (4.5)	
Leukaemia or lymphoma	47 (2.7)	
Head	61 (3.5)	
Other	160 (9.2)	
**Comorbidity (numbers), *n* (%)**		15 (0.9)
0	698 (40.5)	
1	646 (37.5)	
2	287 (16.6)	
≥3	93 (5.4)	
**Current oncology treatment: yes, *n* (%)**		
Chemotherapy	715 (41.4)	13 (0.7)
Radiotherapy	89 (5.2)	14 (0.8)
Hormonal treatment	175 (10.1)	14 (0.8)
Other treatment	97 (5.6)	14 (0.8)
No treatment	700 (40.6)	13 (0.7)
**Current medication: yes, *n* (%)**		
Opioids	1012 (59.3)	33 (1.9)
Non-opioid analgesics	808 (47.4)	35 (2.0)
Corticosteroids	782 (45.8)	30 (1.7)
Laxatives	828 (48.6)	34 (2.0)
Antiemetics	681 (40.2)	43 (2.5)
Sedatives/anxiolytics	526 (30.9)	37 (2.1)
Antidepressants	281 (16.5)	37 (2.1)
**Treatment setting, *n* (%)**		48 (2.8)
Inpatients	365 (21.6)	
Outpatients (day care)	1026 (60.7)	
Home care	300 (17.7)	
**Place of care, *n* (%)**		25 (1.4)
Oncology department	592 (34.5)	
Hospital palliative care unit	788 (46.0)	
Other hospital department	20 (1.2)	
Hospice	144 (8.4)	
Nursing home	15 (0.9)	
Primary care setting/home	155 (9.0)	
**Survival (*n* patients = 1739)**		
**Still alive at the end of the study period, or survival status unknown, *n* (%)**	649 (37.3)	
**Deceased during follow-up, *n* (%)**	1090 (62.7)	
Verified date of death (*n* patients = 1063)^*^		
**Survival in days from inclusion, *n* (%)**		
<30 days	161 (15.1)	
30–89 days	309 (29.1)	
90–149 days	187 (17.6)	
150–180 days	67 (6.3)	
>180 days	339 (31.8)	

Abbreviations: SD, standard deviation; AU, Australia; WE, Western Europe; EE, Eastern Europe; AM, America; NE, Northern Europe; SE, Southern Europe; ME, Middle Europe

Percentages may not sum to 100 due to rounding.

* A date of death was not registered for 27 out of 1090 patients that were reported dead during follow-up.

Of the total sample, 1090 (62.7%) people were reported dead during the follow-up period. A confirmed date of death was not available for 27 participants; survival length for the 1063 people with a verified date of death is categorized in *Table 1* and shows that around 68% died within six months from inclusion.

### Prospective analysis: Quality of life and symptom intensity over time

**Fig 1:**
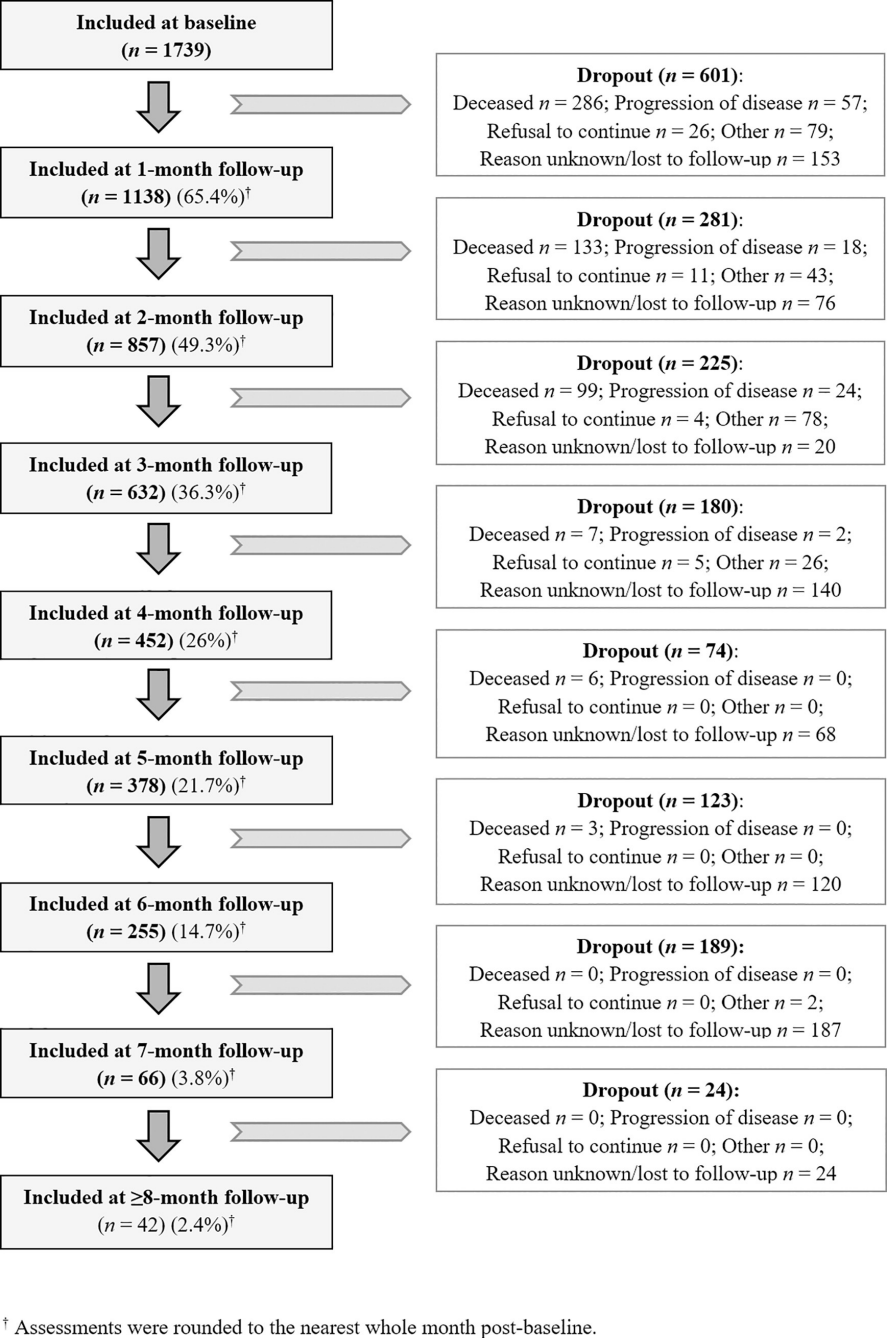
Flow diagram of participants throughout the course of the study.

The sample decreased from 1739 participants at baseline to 1138 (65.4%) at month 1, 857 (49.3%) at month 2, 632 (36.3%) at month 3, 452 (26%) at month 4, 378 (21.7%) at month 5, 255 (14.7%) at month 6, 66 (3.8%) at month 7, and 42 (2.4%) at month ≥8; thus, three-quarters of the sample had dropped out at month 4 (*Fig 1*).

At inclusion (baseline), patients’ EORTC QLQ-C15-PAL estimated mean scores were 51.18 for overall quality of life (QoL), 65.45 for emotional functioning (EF) and 61.77 for physical functioning (PF). No significant differences between baseline and subsequent QoL, EF and PF mean scores were found (*Table 2*). Participants’ mean score for pain was significantly lower, indicating less pain, at month 1 (37.33) compared with baseline (41.88); pain mean scores at the other months were not significantly different from the baseline score (*Table 2*). Mean scores for nausea/vomiting were significantly lower compared with baseline (20.06) at month 4 (15.09), 5 (12.81), 6 (13.65), 7 (13.02) and ≥8 (12.68), suggesting a downward trend which was confirmed by a Mann-Kendal trend test (*p* < .001) (not shown in table). For insomnia, participants scored significantly lower than baseline (33.25) at month 2 (27.91), 3 (27.57) and 4 (26.26). Estimated mean scores for appetite loss were significantly lower than baseline (36.89) at month 3 (30.03) and 5 (29.64). Constipation mean scores were lower at month 3 (21.81) and 4 (21.64) compared to baseline (28.56). Mann-Kendal tests did, however, not reveal statistically significant trends for these symptoms. For dyspnea (baseline mean score 27.17) and fatigue (baseline mean score 52.20), there were no significant differences with baseline at any time point.

**Table 2:** Prospective analysis: Quality of life and symptom intensity over time as assessed by the EORTC QLQ-C15-PAL in people with cancer receiving palliative care.

	Time^§^								
	Baseline (*Ref*) (*n* patients = 1739)^†^	Month 1 (*n* patients = 1138)^†^	Month 2 (*n* patients = 857)^†^	Month 3 (*n* patients = 632)^†^	Month 4 (*n* patients = 452)^†^	Month 5 (*n* patients = 378)^†^	Month 6 (*n* patients = 255)^†^	Month 7 (*n* patients = 66)^†^	Month ≥8 (*n* patients = 42)^†^
	Estimated mean (95% CI)	Estimated mean (95% CI)	Estimated mean (95% CI)	Estimated mean (95% CI)	Estimated mean (95% CI)	Estimated mean (95% CI)	Estimated mean (95% CI)	Estimated mean (95% CI)	Estimated mean (95% CI)
**Overall QoL**^**±**^	51.18 (47.09;55.26)	52.25 (48.07;56.43)	53.81 (49.55;58.08)	52.30 (47.93;56.66)	53.09 (48.50;57.68)	52.69 (47.99;57.39)	51.11 (46.15;56.08)	51.46 (45.59;57.34)	49.80 (43.42;56.19)
**Emotional functioning**^**±**^	65.45 (57.34;73.57)	66.70 (58.56;74.84)	67.35 (59.18;75.52)	67.11 (58.92;75.30)	67.48 (59.24;75.72)	67.97 (59.71;76.24)	66.59 (58.24;74.95)	69.03 (60.37;77.69)	65.17 (56.26;74.09)
**Physical functioning**^**±**^	61.77 (54.06;69.48)	59.36 (51.59;67.14)	59.90 (52.05;67.75)	56.61 (48.72;64.49)	59.41 (51.41;67.41)	58.82 (50.73;66.90)	57.87 (49.57;66.17)	61.68 (52.97;70.40)	58.45 (49.14;67.76)
**Symptoms**^**±**^									
Pain	41.88 (36.87;46.90)	37.33* (32.23;42.42)	37.62 (32.44;42.80)	38.96 (33.71;44.20)	37.60 (32.16;43.03)	40.63 (35.09;46.18)	39.54 (33.69;45.40)	36.24 (29.60;42.88)	43.31 (35.96;50.66)
Fatigue	52.20 (45.50;58.89)	51.72 (44.99;58.46)	49.38 (42.60;56.17)	51.16 (44.33;57.99)	50.28 (43.35;57.22)	49.77 (42.77;56.78)	52.19 (45.01;59.38)	51.41 (43.65;59.17)	52.28 (44.00;60.57)
Nausea/vomiting	20.06 (15.13;24.99)	20.05 (15.08;25.02)	18.24 (13.22;23.26)	16.86 (11.81;21.91)	15.09** (9.99;20.18)	12.81** (7.71;17.91)	13.65** (8.45;18.85)	13.02** (7.41;18.62)	12.68* (6.53;18.82)
Dyspnea	27.17 (20.65;33.69)	28.44 (21.87;35.01)	26.87 (20.24;33.50)	30.86 (24.19;37.54)	27.58 (20.79;34.36)	28.31 (21.47;35.14)	29.20 (22.19;36.21)	31.17 (23.59;38.75)	29.86 (21.68;38.03)
Insomnia	33.25 (28.24;38.25)	29.61 (24.56;34.66)	27.91** (22.78;33.04)	27.57** (22.37;32.77)	26.26** (20.87;31.65)	28.37 (22.88;33.86)	28.58 (22.78;34.37)	31.90 (25.02;38.78)	25.60 (17.95;33.24)
Appetite loss	36.89 (31.31;42.46)	33.58 (27.94;39.23)	32.23 (26.48;37.99)	30.03** (24.19;35.87)	32.30 (26.22;38.37)	29.64* (23.46;35.83)	33.37 (26.84;39.90)	33.73 (26.07;41.38)	33.17 (24.46;41.87)
Constipation	28.56 (25.80;31.33)	25.49 (22.57;28.41)	25.58 (22.52;28.65)	21.81** (18.56;25.06)	21.64** (17.96;25.31)	23.54 (19.64;27.43)	23.30 (18.93;27.67)	24.80 (18.89;30.70)	28.07 (21.30;34.83)

Abbrevations: QoL, quality of life; CI, confidence interval

^**§**^ Linear mixed models were analyzed with repeated measures on patients nested in hospitals, and hospitals in countries.

Bonferroni-Holm adjustment was applied to all pairwise comparisons.

^**±**^ Scores were linearly converted into a 0–100 scale according to the EORTC guidelines, with higher values representing better quality of life, better functioning and higher symptom severity.

** Estimated mean score differed significantly from baseline value using an experiment-wise error rate (EER) of 0.01.

* Estimated mean score differed significantly from baseline value using an experiment-wise error rate (EER) of 0.05.

^†^ ≥1 assessment per patient possible because assessments were rounded to the nearest whole month.

### Retrospective analysis: Quality of life and symptom intensity over time towards death

**Fig 2:**
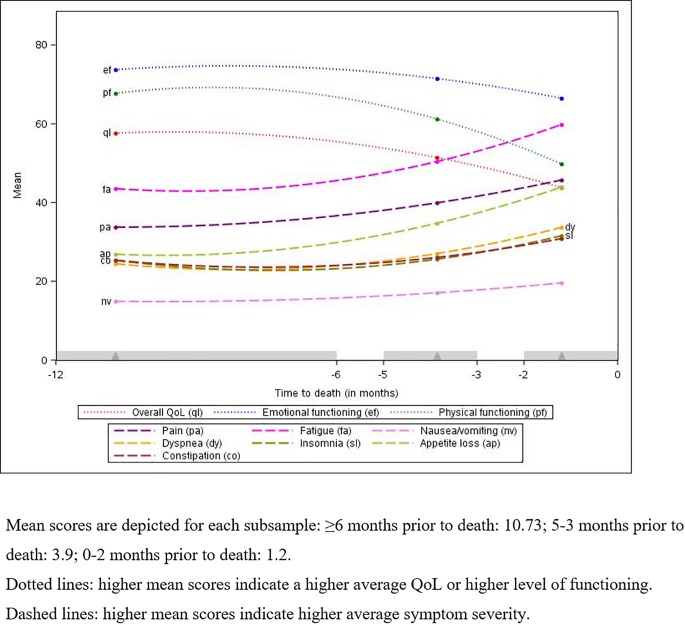
Retrospective analysis: Quality of life and symptom intensity over time towards death.

*Table 3* shows estimated mean scores for QoL, EF and PF obtained from three cross-sectional subsamples at different stages of illness (time before death, group 1: ≥6 months, group 2: 5–3 months, group 3: 2–0 months). For QoL and PF, mean scores of participants 2–0 months prior to death (group 3) were significantly lower than mean scores of those 5–3 months prior to death (group 2), and mean scores of the latter group were significantly lower than mean scores of those ≥6 months prior to death (group 1). This suggests that QoL and PF worsened throughout the disease trajectory towards death. For EF, a significant decline was only found when comparing those 5–3 months prior to death (group 2) with those 2–0 months prior to death (group 3). *Fig 2* shows QoL, EF and PF for the different subsamples.

**Table 3:** Retrospective analysis: Quality of life and symptom intensity over time towards death.

	Time to death (in months)^§^					
	Period 1 (≥6) (*n* patients = 369)^†^	Period 2 (5–3) (*n* patients = 442)^†^	Period 3 (2–0) (*n* patients = 675)^†^	Δ Period 2 and period 1	Δ Period 3 and period 2	Δ Period 3 and period 1
	Estimated mean (95% CI)	Estimated mean (95% CI)	Estimated mean (95% CI)	Mean difference (*p*-value)	Mean difference (*p*-value)	Mean difference (*p*-value)
**Overall QoL**^**±**^	57.62 (53.32;61.92)	51.44 (47.19;55.68)	43.83 (39.60;48.07)	-6 (< .001)	-8 (< .001)	-14 (< .001)
**Emotional functioning**^**±**^	73.74 (68.45;79.03)	71.49 (66.25;76.72)	66.49 (61.27;71.72)	-2 (.078)	-5 (< .001)	-7 (< .001)
**Physical functioning**^**±**^	67.73 (62.11;73.34)	61.20 (55.71;66.70)	49.82 (44.35;55.29)	-7 (.005)	-11 (< .001)	-18 (< .001)
**Symptoms**^**±**^						
Pain	33.77 (29.28;38.26)	39.94 (35.49;44.39)	45.72 (41.28;50.16)	+6 (.002)	+6 (.003)	+12 (< .001)
Fatigue	43.54 (38.62;48.46)	50.42 (45.56;55.28)	59.80 (54.95;64.65)	+7 (< .001)	+9 (< .001)	+16 (< .001)
Nausea/vomiting	14.93 (10.61;19.25)	17.10 (12.88;21.32)	19.61 (15.44;23.78)	+2 (.247)	+3 (.156)	+5 (.016)
Dyspnea	24.49 (18.27;30.71)	27.05 (20.90;33.20)	33.72 (27.59;39.85)	+3 (.085)	+7 (< .001)	+9 (< .001)
Insomnia	25.38 (20.39;30.37)	25.63 (20.73;30.52)	31.57 (26.71;36.43)	+0 (.901)	+6 (.002)	+6 (.003)
Appetite loss	26.89 (21.63;32.15)	34.76 (29.60;39.91)	43.94 (38.80;49.07)	+8 (< .001)	+9 (< .001)	+17 (< .001)
Constipation	25.33 (21.17;29.48)	26.08 (22.04;30.13)	30.86 (26.80;34.92)	+1 (.593)	+5 (.001)	+6 (.001)

Abbrevations: QoL, quality of life CI, confidence interval

^**§**^ Linear mixed model analyses were performed with repeated measures on patients nested in hospitals, and hospitals in countries.

^**±**^ Scores were linearly converted into a 0–100 scale according to the EORTC guidelines, with higher values representing better quality of life, better functioning and higher symptom intensity.

^†^ ≥1 assessment per patient possible.

Participants 2–0 months prior to death (group 3) were found to have significantly higher mean scores for pain, fatigue and appetite loss than those 5–3 months prior to death (group 2), and those from the latter group were found to have significantly higher mean scores than those ≥6 months prior to death (group 1), indicating an increase in intensity, thus deterioration, of these symptoms over time towards death (*Table 3*). For dyspnea, insomnia and constipation, a significant deterioration was only found when comparing the mean scores of those 5–3 months prior to death (group 2) with the mean scores of those 2–0 months prior to death (group 3), and for nausea/vomiting only when comparing those ≥6 months prior to death (group 1) with those 2–0 months prior to death (group 3).

## Discussion

### Main findings

To our knowledge, this is the first multi-center study to evaluate changes in self-reported quality of life (QoL), emotional functioning (EF), physical functioning (PF), and symptoms (pain, fatigue, nausea/vomiting, dyspnea, insomnia, appetite loss, constipation) over time in a large international sample of people with advanced cancer receiving palliative care. A prospective analysis of the entire study sample showed general stability for QoL, EF, PF and symptoms from baseline throughout the study period (≥8-month follow-up). A retrospective analysis of participants who had passed away during follow-up revealed a significant deterioration towards death for QoL, PF, pain, fatigue and appetite loss when comparing cross-sectional subsamples of patients ≥6 months prior to death with those 5–3 months prior to death, and those 5–3 months prior to death with 2–0 months prior to death. EF, dyspnea, insomnia and constipation only showed significant deterioration when comparing those 5–3 months prior to death with those 2–0 months prior to death.

A possible explanation for the consistency of QoL, EF, PF and symptom intensity over time in the prospective analysis is that cancer in general tends to follow a trajectory of a long period of clinical stability. This reflects the well-known trajectory representing the typical course of cancer proposed by Lynn and Adamson (2003) [9] according to which individuals often maintain comfort and relatively normal functioning for a substantial time, and only show a rapid decline in the final weeks before death. Moreover, intensification of palliative care to alleviate suffering and enhance comfort may have prevented further deterioration of QoL and slowed or stabilized symptom progression over time.

The prospective finding that QoL, functioning and symptoms were stable over time did not exclude the possibility of symptoms to get worse or become harder to control as death approaches (i.e., the typical period of evident decline near the end of life as described by Lynn and Adamson), because this analysis also included patients who were still alive at the end of the follow-up period. Our retrospective analysis revealed a different picture, suggesting that deterioration in QoL, PF and three prominent symptoms (pain, fatigue, appetite loss) did not only occur at the end of life (in the terminal phase), but became noticeable already relatively early, around 5–3 months before death.

A striking finding was that EF did not significantly decrease when comparing those ≥6 months prior to death with those 5–3 months prior to death but did seem to deteriorate substantially in the last few months (i.e. when comparing those 5–3 months prior to death with those 2–0 months prior to death). It is possible that patients experience a coping shift or a delayed emotional response to acknowledging the physical changes or challenges that are likely to become more pronounced as the disease progresses, such as reduced energy and poor appetite. It could also have been a result of the increasing number of stressful circumstances that may be experienced by individuals who are facing death in the near future (e.g., uncertainty, numerous losses and adjustments, anticipatory grief) [16]. However, emotional changes may occur at different times and intensities throughout the disease trajectory, and cancer patients in palliative care are known to be a vulnerable population at risk of mental health problems including anxiety and depressive symptoms [17–19]. Since psychological distress in cancer patients may lead to adverse outcomes such as poorer QoL and survival, timely recognition and management of distress is essential.

Comparing our results directly to data from other studies is difficult because of the wide variety of population characteristics, data collection methods and instruments used across studies. Our findings are, however, generally consistent with available evidence suggesting that QoL, functioning and symptom distress change at different rates at different points in time, and that decline tends to spiral downwards more rapidly in the last months or weeks of life (‘terminal drop’) [20–26]. Additionally, it is interesting to note that pain, fatigue and appetite loss not only were the three symptoms starting to deteriorate most early, but were also the most severe symptoms (i.e., highest mean scores) in all three different periods of time before death. This finding is not completely unexpected, since it is known from previous research that pain, fatigue and appetite loss are among the most debilitating and commonly reported symptoms in advanced cancer [3,27–30]. Nausea/vomiting was relatively the least severe symptom, a result that is consistent with previous research showing that nausea and vomiting are generally less bothersome and less frequently occurring symptoms in patients with advanced disease than other symptoms such as pain, fatigue and dyspnea [31], also in palliative care populations [20,32,33].

### Strengths and limitations

The EPCCS study is the largest international, longitudinal study in a palliative care cancer population of which we are aware. Strengths of this research include the prospective design, the sample size and the inclusion of patients from multiple centres, which made it possible to follow QoL, EF, PF and symptoms over an extended period of time, in a considerable number of vulnerable people in Europe and beyond. The linear mixed model procedure adopted in this study is a powerful approach as it takes account of repeated measurement and clustering effects at both hospital and country level. Despite these strengths, our study also has limitations which need to be acknowledged. The first concerns the representativeness of the sample. The EPCCS study’s main report [10] showed that there is large variation in the organization and delivery of palliative care services and in patient characteristics (e.g. primary tumor sites, stages and treatment regimens) across Europe. Clear population criteria will be essential in future research to facilitate the comparison of results across different studies and countries [10]. A second notable issue is that data were relatively sparse in the last months of the study. Patient attrition is an inherent difficulty of longitudinal studies, especially in palliative care where drop out due to deterioration or death is very likely. Third, it is possible that participants with the worst levels of functioning were not included in the study, which may have resulted in an underestimation of the QoL and symptom experience.

### Implications for research and practice

The findings of our study indicate that optimization of QoL, functioning and symptom relief remain challenges to healthcare providers involved in palliative cancer care and becomes more difficult to achieve as the illness progresses towards death. There is a need for systematic and standardised screening of QoL, functioning and physical as well as psychological symptoms to become an integral part of clinical routine during the disease trajectory. This could help determine when palliative needs to be strengthened and guide the care for these individuals, which is important since physicians tend to underestimate the severity of symptoms [34]. Moreover, routine standardised self-report of symptoms may improve patient-physician communication [35]. Additional studies should investigate which strategies of screening are most effective [36].

The present study was explorative in nature and did not assess factors associated with QoL, functioning and symptoms over time. It would be interesting for future research to investigate what characterizes patients with different levels of symptomatology (e.g. low, medium, high) at different time points before death. From a clinical point of view, it might be valuable to distinguish between symptoms that are less ore more difficult to control by medication as death comes closer.

### Conclusions

A prospective analysis of a large international sample of palliative care cancer patients showed that self-reported QoL, functioning and cancer-related symptoms remained stable from inclusion (baseline) over time throughout the study duration, where a retrospective analysis revealed that, although deterioration accelerated as death approached, this was already apparent before the terminal phase. Our findings further support the importance of early symptom detection and treatment in this population, and the need for future research to look at characteristics of patients with lower or higher symptom burden at different time points towards death.
